# A new species of the genus *Castoponera* (Araneae, Corinnidae) from Sarawak, Borneo, with comparison to a related species

**DOI:** 10.3897/zookeys.596.8525

**Published:** 2016-06-07

**Authors:** Takeshi Yamasaki, Yoshiaki Hashimoto, Tomoji Endo, Fujio Hyodo, Takao Itioka

**Affiliations:** 1 Graduate School of Science and Engineering, Tokyo Metropolitan University, 1-1 Minami-osawa, Hachioji-shi, Tokyo 192-0397, Japan; 2 Institute of Natural and Environmental Sciences, University of Hyogo/Museum of Nature and Human Activities, Hyogo, Sanda, Hyogo 669-1546, Japan; 3 School of Human Science, Kobe College, Nishinomiya, Hyogo 662-8505, Japan; 4 Research Core for Interdisciplinary Sciences, Okayama University, Okayama 700-8530, Japan; 5 Graduate School of Human and Environment Studies, Kyoto University, Kyoto 606-8501, Japan

**Keywords:** Castianeirinae, taxonomy, myrmecomorphy, Southeast Asia

## Abstract

A new species of the genus *Castoponera* Deeleman-Reinhold, 2001, *Castoponera christae* **sp. n.**, is described here. The species is closely related to *Castoponera lecythus* Deeleman-Reinhold, 2001, but can be distinguished by the structures of the male palp and the female genitalia.

## Introduction

The genus *Castoponera* Deeleman-Reinhold, 2001 is endemic to Southeast Asia, and is currently comprised of three species (Deeleman-Reinhold 2001; World Spider Catalog 2016). From Borneo, two species, *Castoponera scotopoda* Deeleman-Reinhold, 1993 and *Castoponera lecythus* Deeleman-Reinhold, 2001, have been recorded. *Castoponera* species closely resemble Ponerinae ants and they commonly occur on the forest floor. The morphological resemblance to ants is known as myrmecomorphy and is a common phenomenon in the Corinnidae (Cushing 1997, 2012; Haddad 2013; Raven 2015; Reiskind 1969).

Our group has conducted several investigations in Borneo to reveal the association between ant-mimicking spiders and ants. Although the Corinnidae fauna in Southeast Asia has been comprehensively reviewed by Deeleman-Reinhold (1993, 2001), our investigations have resulted in the discovery of an undescribed corinnid species. We here describe it, in comparison with the closely related species, *Castoponera lecythus*.

## Materials and methods

**Figure 1.**
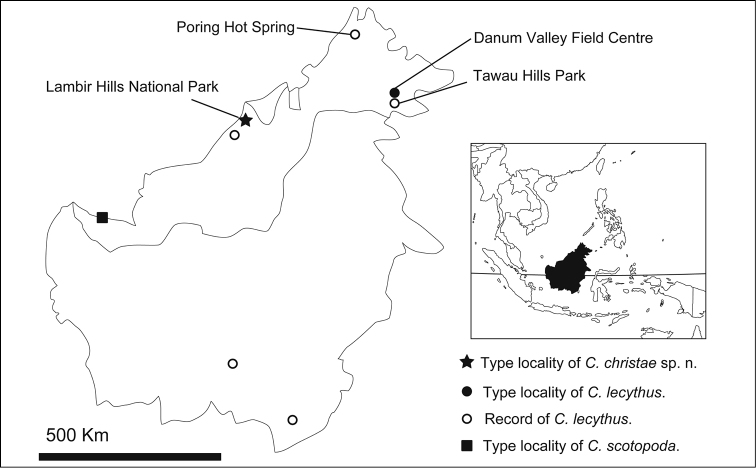
Study sites and distribution of *Castoponera* species on Borneo (modified from Deeleman-Reinhold 2001).

Specimens examined here were collected from the forest floors in Danum Valley Field Centre, Tawau Hills Park and Poring Hot Spring, Sabah, and Lambir Hills National Park, Sarawak, Borneo (Fig. 1). Collected spiders were preserved in 75% ethanol. The morphology was examined using a Nikon SMZ1270 microscope, and specimens were sorted and identified on the basis of descriptions in Deeleman-Reinhold (2001). Multi-focused montage images were produced using Helicon Focus ver. 4.2.9 from several series of source images. The habitus images were obtained using a Canon EOS 60D camera with a Canon MP-E 65mm macro lens and the images of the male palp and female genitalia were obtained by the same camera attached to a Nikon AZ100 microscope.

Methodology and terminology for the description follow Deeleman-Reinhold (2001). The leg spination of each segment is described as a row of spines from proximal to distal parts on each side (dorsal, ventral, prolateral and retrolateral sides). However, recognition of spine position on distal part of metatarsi III and IV was very difficult due to the narrow segments. For the distal spines on these segments the total number of spines is given, without positional information. For the width of the eye region, the width of posterior eye row was measured. All measurements are given in millimeters. Abbreviations used in the present paper are as follows: ALE, anterior lateral eyes; AME, anterior median eyes; d, dorsal; pl, prolateral; PLE, posterior lateral eyes; PME, posterior median eyes; rl, retrolateral; v, ventral.

The holotype designated here is deposited in the Forest Research Centre, Sarawak, Malaysia (FRCS), and the paratypes in FRCS and the Museum of Nature and Human Activities, Hyogo, Japan (MNHAH).

## Taxonomy

### *Castoponera* Deeleman-Reinhold, 2001

#### Castoponera christae

Taxon classificationAnimaliaAraneaeCorinnidae

Yamasaki sp. n.

http://zoobank.org/F527D81F-E1D1-4FC9-A0C2-69C5333D96BA

Figs 2–8 , 9–14 , 15–18

##### Type material.

**Holotype male** (FRCS; LCo20090226 Itioka), Sungai Liku (Liku River), 4°14'N, 114°03'E, Lambir Hills National Park, Sarawak, Borneo, 29-II-2009, T. Itioka leg. **Paratypes**: 1 female (FRCS; LCo20070822-AMS2), same locality as the holotype, 22-VIII-2007, Y. Hashimoto & T. Endo leg.; 1 male (MNHAH; LCo20140331-HYO1), same locality as the holotype, 31-III-2014, F. Hyodo leg.

##### Diagnosis.

In males, *Castoponera christae* sp. n. is distinguishable from *Castoponera ciliata* (Deeleman-Reinhold, 1993) and *Castoponera scotopoda* by the long embolus (Figs 6, 15, cf. figs 445, 449 in Deeleman-Reinhold 2001), and from *Castoponera lecythus* by the tapering distal region of the bulb: lateral margins are more or less parallel in *Castoponera lecythus* (Figs 6, 15 vs. Figs 23, 32). In females, *Castoponera christae* sp. n. is distinguishable from *Castoponera ciliata* and *Castoponera scotopoda* by the long and curved insemination ducts (Figs 14, 18, cf. figs 448, 451 in Deeleman-Reinhold 2001), and from *Castoponera lecythus* by the position of the copulatory opening on the copulatory atrium, the position of insemination duct where it joins the bursa, and the rounded shape of the bursa (Figs 13–14, 17–18 vs. Figs 30–31, 34–35).

**Figures 2–8.**
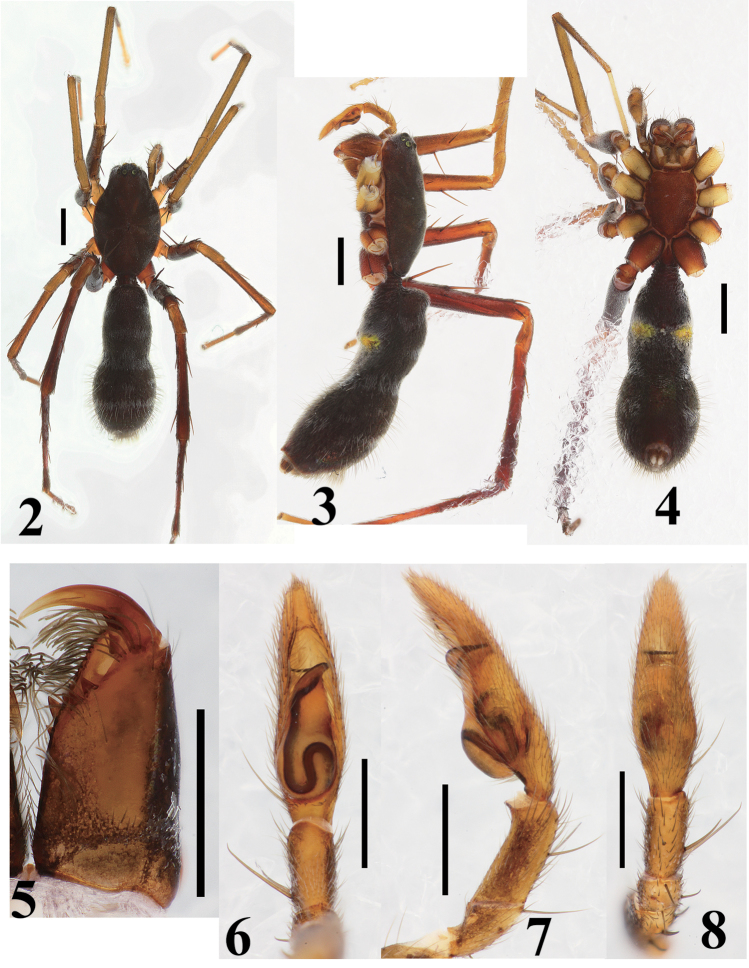
*Castoponera christae* sp. n., male. **2** habitus, dorsal view **3** habitus, lateral view **4** habitus, ventral view **5** chelicera and fang, ventral view **6** palp, ventral view **7** palp, retrolateral view **8** palp, dorsal view. Scales: 1.0 mm (**2–4**), 0.5 mm (**5–8**).

**Figures 9–14.**
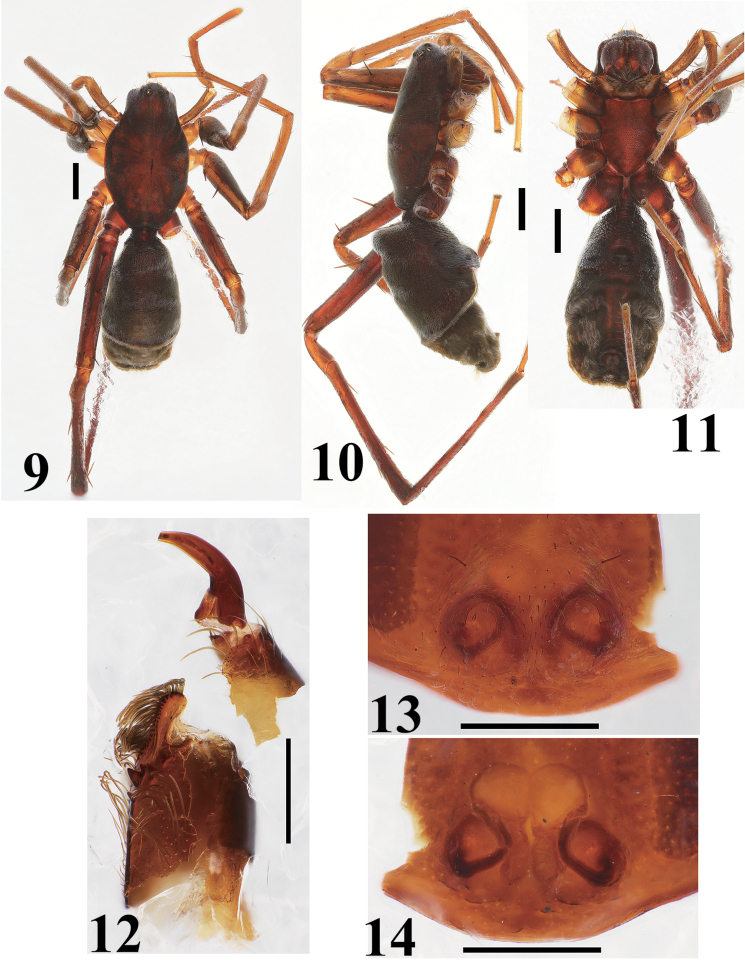
*Castoponera christae* sp. n., female. **9** habitus, dorsal view **10** habitus, lateral view **11** habitus, ventral view **12** chelicera and fang, ventral view **13** epigyne, ventral view **14** internal structures of genitalia, dorsal view. Scales: 1.0 mm (**9–11**), 0.5 mm (**12–14**).

**Figures 15–18.**
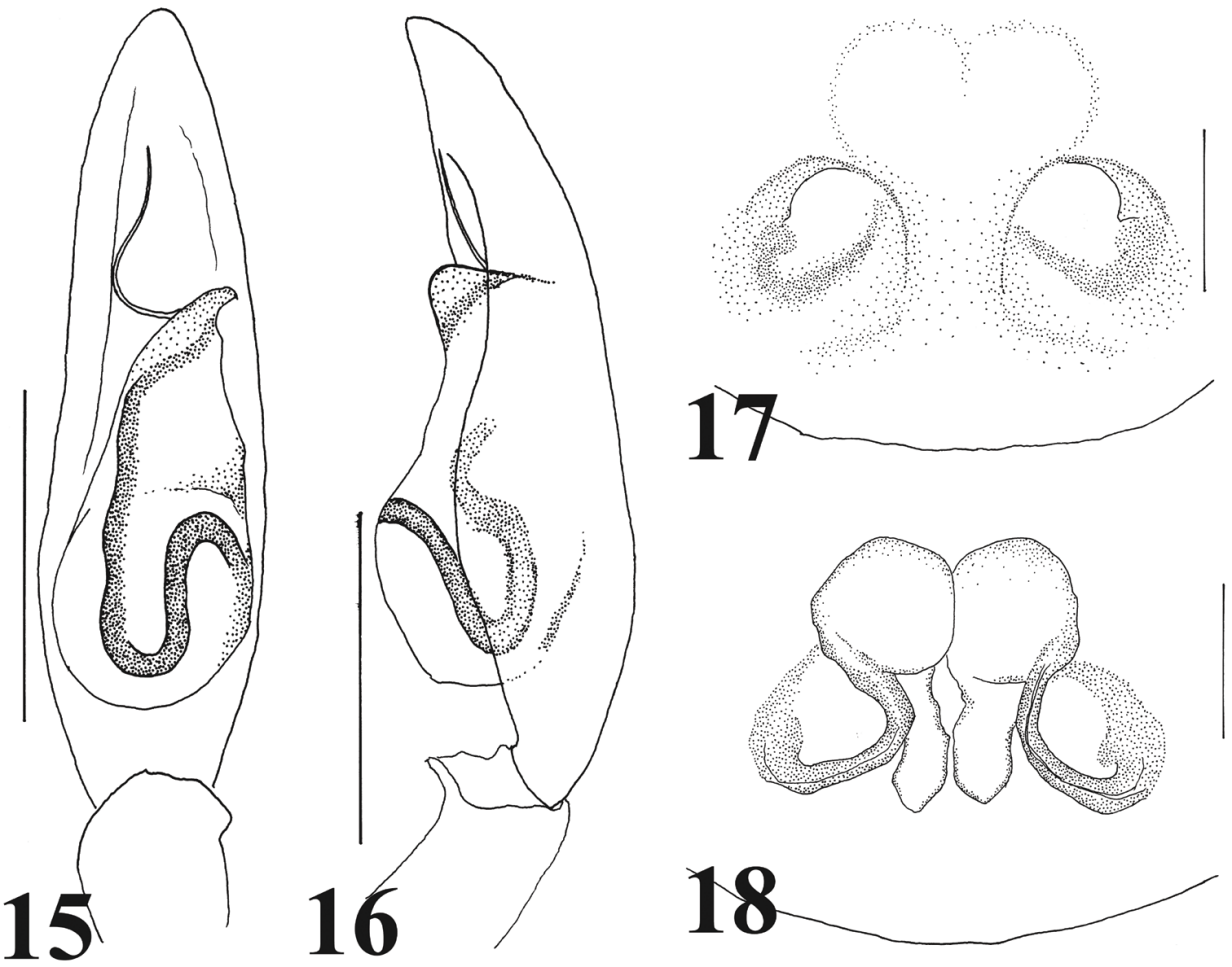
*Castoponera christae* sp. n. **15** male palp, ventral view **16** male palp, retrolateral view **17** epigyne, ventral view **18** internal structures of genitalia. Scales: 0.5 mm (**15–16**), 0.25 mm (**17–18**).

**Figures 19–25.**
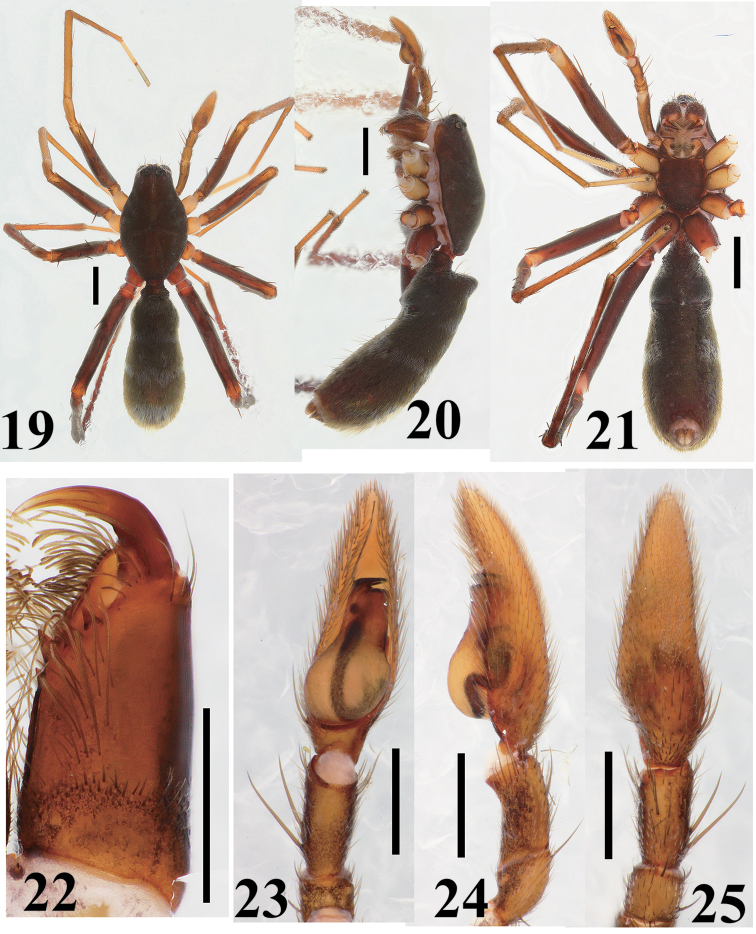
*Castoponera lecythus*, male. **19** habitus, dorsal view **20** habitus, lateral view **21** habitus, ventral view **22** chelicera and fang, ventral view 23 palp, ventral view **24** palp, retrolateral view **25** palp, dorsal view. Scales: 1.0 mm (**19–21**), 0.5 mm (**22–25**).

**Figures 26–31.**
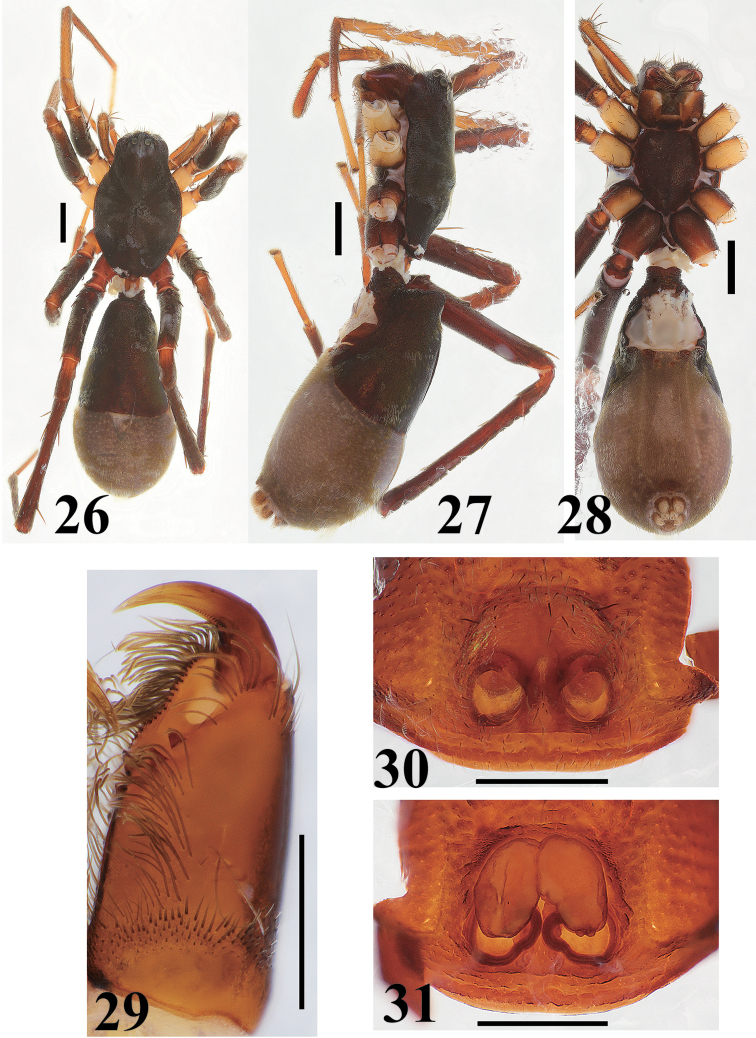
*Castoponera lecythus*, female. **26** habitus, dorsal view **27** habitus, lateral view **28** habitus, ventral view **29** chelicera and fang, ventral view **30** epigyne, ventral view **31** internal structures of genitalia, dorsal view. Scales: 1.0 mm (**26–28**), 0.5 mm (**29–31**).

**Figures 32–35.**
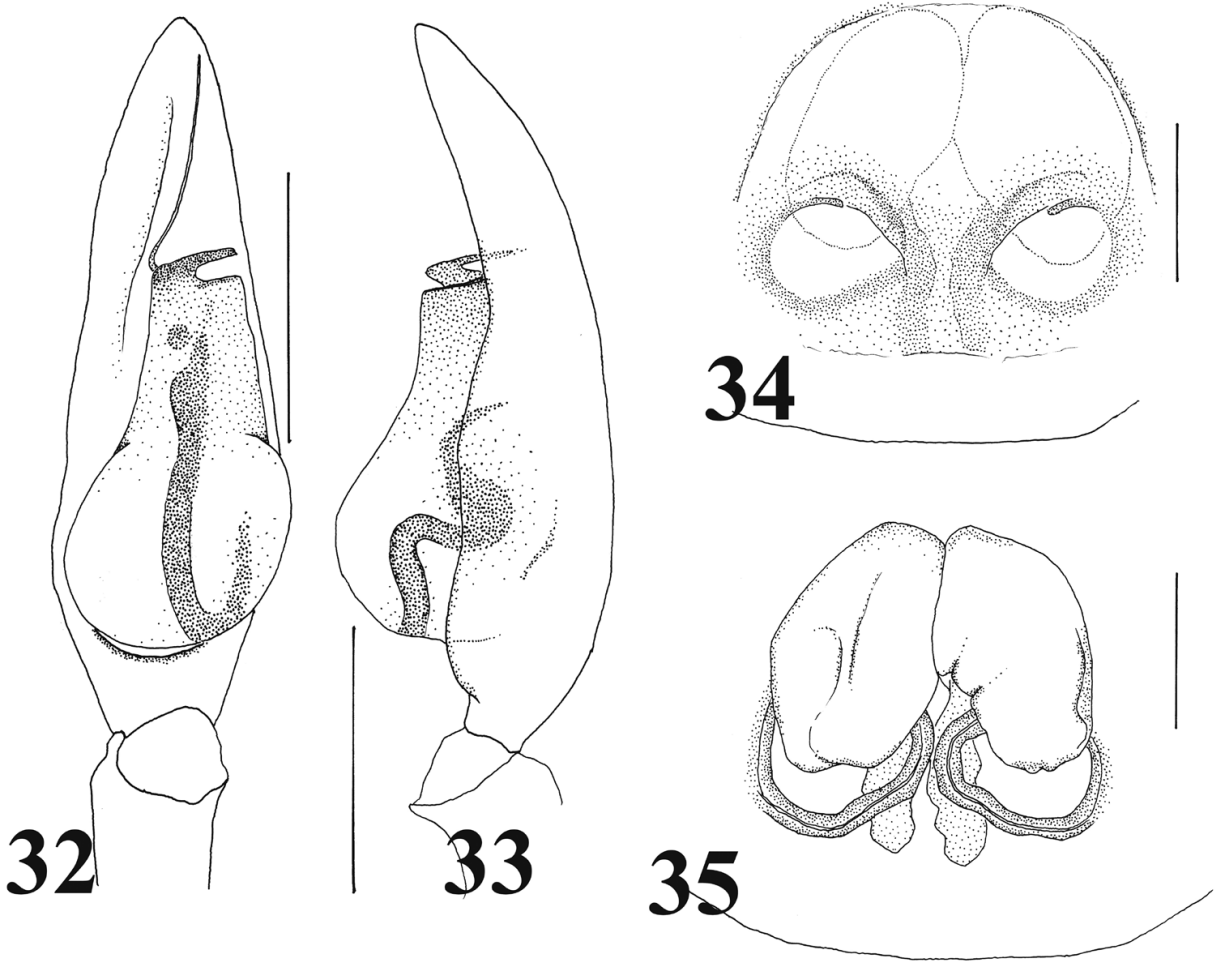
*Castoponera lecythus*. **32** male palp, ventral view **33** male palp, retrolateral view **34** epigyne, ventral view **35** internal structures of genitalia, dorsal view. Scales: 0.5 mm (**32–33**), 0.25 mm (**34–35**).

##### Measurements

(holotype male/paratype female). Total length 7.4/8.5. Carapace length 3.07/4.20; width 1.87/2.43; height 0.97/1.20. Clypeus height 0.28/0.37. Eye size: AME 0.18/0.21; ALE 0.12/0.15; PME 0.14/0.18; PLE 0.14/0.18. Width of eye region 0.68/0.98. Distance between PMEs 0.13/0.15. Abdomen length 4.70/8.80; width 1.67/2.33.

##### Male

(Figs 2–5). Carapace oval, with granulated surface (Fig. 2). Chelicera with three promarginal and two retromarginal teeth on fang furrow (Fig. 5). Retrocoxal hymen obviously smaller than ALE, approximately 0.06 mm in diameter. Pedicel wrapped in tube-like sclerite extending from abdomen (Figs 3–4). Abdomen slender pear-shaped, constricted at middle part; entire surface strongly sclerotized (Fig. 2).

Male palp (Figs 6–8, 15–16). Cymbium slender (Fig. 8). Bulb slender teardrop-shaped, including tapering anterior part and globular posterior part (Figs 6, 15); distal part curved toward retrolateral side (Figs 6, 15). Sperm duct beginning at retrolateral surface of bulb, strongly curving once at retrolateral and twice at ventral surfaces (Figs 6–7, 15–16), then extending to embolus (Figs 6, 15). Embolus very slender, strongly curved on horizontal plan against longitudinal axis (Figs 6, 15).

Leg spination. Femur I 1-1-1d, 1pl; tibia I 2-2-2v; metatarsus I 2-2v; femur II 1-1-1d, 1pl; tibia II 2-2-2v; metatarsus II 2-2v; femur III 1-1-1d, 1-1pl, 1-1rl; tibia III 1d, 1-2v, 1-1pl, 1-1rl; metatarsus III 2-2v, 1-1pl, 1-1rl with 5 distal spines; femur IV 1-1-1d, 1-1pl, 1-1rl; tibia IV 1d, 1-2-2v, 1-1pl, 1-1rl; metatarsus IV 2-2v, 1-1pl, 1-1rl with 5 distal spines.

Coloration and setation (Figs 2–4). Carapace dark brown, covered with short fine setae; anterior surface near eye region covered with white plumose setae. Chelicera brown; anterior surface sparsely covered with long gray setae and short transparent setae; promargin of fang furrow densely fringed with long thick setae whose surfaces are rough (Fig. 5). Labium, maxilla, sternum brown. Legs covered with black setae, black plumose setae and transparent plumose setae; plumose setae sparse in tarsi; coxae I, II and III brownish cream; coxa III more darker than I and II; coxa IV brown; trochanters almost same coloration as in coxae; femora light brown, tinged with black in femora I, II and III; patellae yellowish cream to brownish cream; tibiae I and II grayish yellow, III and IV light brown; metatarsi almost same coloration as in tibiae; tarsus I cream, tarsi II and III brownish cream, tarsus IV light brown. Pedicel dark brown. Abdomen blackish brown; entire surface covered with white fine plumose setae, posterior surface additionally covered with long setae; thick white plumose setae forming following markings: a pair of patches and transverse band on anterior dorsum, transverse band encircling abdominal constriction, two or three patches and transverse band on posterior dorsum; posterior end bearing tuft of white long plumose setae.

##### Female

(Figs 9–12). Almost same as in male, except for abdomen. Abdomen without distinct constriction; anterior half covered with strongly sclerotized surface (Figs 10–11).

Female genitalia (Figs 13–14, 17–18). Copulatory atrium round; copulatory opening located at outer margin of atrium (Figs 13, 17). Insemination duct curving, connecting to outer margin of bursa (Figs 14, 18). Bursa round, accompanying slender spermatheca on posterior margin (Figs 14, 18).

Leg spination. Femur I 1-1-1d, 1pl; tibia I 2-2-2v; metatarsus I 2-2v; femur II 1-1-1d, 1pl; tibia II 2-2-2v; metatarsus II 2-2v; femur III 1-1-1d, 1-1pl, 1-1rl; tibia III 1d, 1-2v, 1-1pl, 1-1rl; metatarsus III 2-2v, 1-1pl, 1-1rl with 5 distal spines; femur IV 1-1-1d, 1-1pl, 1-1rl; tibia IV 1d, 1-1-2v, 1-1pl, 1-1rl; metatarsus IV 2-2v, 1-1pl, 1-1rl with 5 distal spines.

Coloration and setation. Almost same as in male.

##### Etymology.

The specific epithet is a patronym in honor of Dr. Christa L. Deeleman-Reinhold, who has made great contributions in studies of corinnid spiders from Southeast Asia.

##### Distribution.

Lambir Hills National Park, Sarawak.

##### Remarks.

For the female paratype, some morphological characters of the abdomen were not observed because the specimen had once been dried and the soft part of the abdomen was shrunken. However, the sclerotized parts of the abdomen and the genitalia have been well preserved and the identification is possible on the basis of these characters.

*Castoponera christae* sp. n. is closely related to *Castoponera lecythus*. The male of *Castoponera christae* sp. n. can be distinguished from the male of *Castoponera lecythus* by the medially constricted abdomen (Figs 2–4 vs. Figs 19–21), shape of apical part of the bulb and route of the sperm duct running on the surface of the bulb (Figs 6–7, 15–16 vs. Figs 23–24, 32–33). Additionally, among our specimens of each species, the posterior bulb of *Castoponera christae* sp. n. is more swollen than that of *Castoponera lecythus*. In the females it is relatively difficult to distinguish the species using superficial characters. However, the internal genitalic structures are clearly distinct (Figs 14, 18 vs. Figs 31, 35).

#### Castoponera lecythus

Taxon classificationAnimaliaAraneaeCorinnidae

Deeleman-Reinhold, 2001

Figs 19–25 , 26–31 , 32–35

Castoponera lecythus Deeleman-Reinhold, 2001: 314, figs 452–463.

##### Material examined.

1 male (MNHAH; AMS-Hy6), Danum Valley Field Centre, 4°58'N, 117°48'E, Sabah, Borneo, 18-XII-2006, Y. Hashimoto leg.; 1 female (MNHAH; AMS3), same locality, 9-I-2008, Y. Hashimoto & T. Endo leg.; 1 female, Tawau Hills Park, 4°23'N, 117°53'E, Sabah, Borneo, 17-XI-2009, T. Yamasaki leg.; 1 male, Poring Hot Spring, Kinabalu Park, 6°02'E, 116°42'E, Sabah, Borneo, 12-XI-2010, T. Yamasaki leg.

##### Measurements

(Male: AMS-Hy6/Female: AMS3). Total length 7.4/8.7. Carapace length 3.13/3.45; width 1.87/2.13; height 0.98/1.05. Clypeus height 0.26/0.28. Eye size: AME 0.18/0.20; ALE 0.12/0.13; PME 0.15/0.18; PLE 0.15/0.18. Width of eye region 0.70/0.80. Distance between PMEs 0.13/0.16. Abdomen length 3.10/5.00; width 1.58/2.50.

##### Male

(Figs 19–22). Carapace oval, with granulated surface (Fig. 19). Chelicera with three promarginal and two retromarginal teeth on fang furrow (Fig. 22). Retrocoxal hymen obviously smaller than ALE, approximately 0.05 mm in diameter. Pedicel wrapped in tube-like sclerite extending from abdomen (Figs 19–21). Abdomen slender oval, slightly constricted at anterior part; entire surface strongly sclerotized (Fig. 20).

Male palp (Figs 23–25, 32–33). Cymbium slender (Fig. 25). Bulb teardrop-shaped but retrolateral corner of anterior bulb squarish; posterior part spherical, slightly asymmetrical (Figs 23, 32). Sperm duct beginning at retrolateral surface of bulb, curving twice at retrolateral and once at ventral surfaces, then running directly into embolus through center of bulbal surface (Figs 23–24, 32–33).

Leg spination. Femur I 1-1-1d, 1pl; tibia I 2-2-2v; metatarsus I 2-2v; femur II 1-1-1d, 1pl; tibia II 1-2-2v; metatarsus II 2-2v; femur III 1-1-1d, 1-1pl, 1-1rl; tibia III 1d, 1-2v, 1-1pl, 1-1rl; metatarsus III 2-2v, 1-1pl, 1-1rl, with 5 distal spines; femur IV 1-1-1d, 1-1pl, 1rl; tibia IV 1d, 1-1-2v, 1-1pl, 1-1rl; metatarsus IV 2-1v, 1-1pl, 1-1rl, with 5 distal spines.

Coloration and setation (Figs 19–22). Carapace dark brown, covered with short fine setae; anterior surface near eye region covered with white plumose setae. Chelicera brown; anterior surface sparsely covered with long gray setae and transparent plumose setae; promargin of fang furrow densely fringed with long thick setae whose surfaces are rough (Fig. 22). Labium, maxilla, sternum brown. Legs covered with black setae, black plumose setae and transparent plumose setae; plumose setae sparse in tarsi; coxae I, II and III brownish cream; coxa III darker than I and II; coxa IV brown; trochanters almost same coloration as in coxae; femora brown; patellae I and II yellowish brown, III and IV light brown; tibiae I and II yellowish brown, III light brown, IV brown; metatarsi almost same coloration as in tibiae; tarsi yellowish brown, IV more darker than others. Pedicel dark brown. Abdomen blackish brown; entire surface covered with plumose setae, some white and some light brown, and posterior surface covered with long setae; thick white plumose setae forming following markings: transverse band on anterior dorsum, transverse band encircling middle part, large patch on posterior dorsum; posterior end bearing tuft of long white plumose setae.

##### Female

(Figs 26–29). Almost same as in male, except for abdomen. Abdomen without distinct constriction; anterior half covered with strongly sclerotized surface (Figs 26–28).

Female genitalia (Figs 30–31, 34–35). Copulatory atrium round; copulatory opening located at anterior margin (Figs 30, 34). Insemination duct, curving, connecting to inner margin of posterior bursa (Figs 31, 35). Bursa oval, accompanying slender spermatheca on posterior margin (Figs 31, 35).

Leg spination. Femur I 1-1-1d, 1-1pl; tibia I 1-2-2v; metatarsus I 2-2v; femur II 1-1d, 1pl; tibia II 2-2-2v; metatarsus II 2-2v; femur III 1-1d, 1pl, 1rl; tibia III 1d, 1-2v, 1-1pl, 1-1rl; metatarsus III 2-2v; 1-1pl, 1-1rl, with 5 distal spines; femur IV 1-1-1d, 1-1pl, 1-1rl; tibia IV 1d, 1-1-2v, 1-1pl, 1-1rl; metatarsus IV 2-2v, 1-1pl, 1-1rl, with 5 distal spines.

Coloration and setation (Figs 26–29). Almost same as in male.

##### Distribution.

Danum Valley Feild Centre, Sabah (Deeleman-Reinhold 2001); Tawau Hills Park, Sabah; Poring Hot Spring, Kinabalu Park, Sabah; Niah cave National Park, Sarawak (Deeleman-Reinhold 2001); Kaharian, 2°02S, 113°40'E, SE Kalimantan (Deeleman-Reinhold 2001); Aranio district, SE Kalimantan (Deeleman-Reinhold 2001).

##### Remarks.

We examined 1 male and 1 female collected from the type locality, and these specimens agreed with the original description of *Castoponera lecythus*. For the comparison with *Castoponera christae* sp. n., see Diagnosis and Remarks in *Castoponera christae* sp. n.

### Notes on *Castoponera christae* sp. n. and *Castoponera lecythus*

**Figures 36–37.**
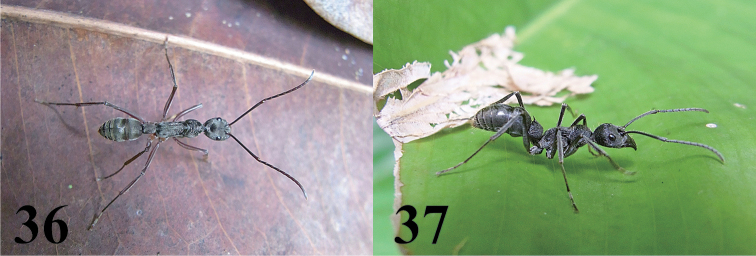
*Diacamma* spp. **36** *Diacamma* sp., Poring Hot Spring, Borneo **37** *Diacamma* sp. Java.

The members of Castianeirinae are considered to be myrmecomorphies (Cushing 1997; Deeleman-Reinhold 2001). In the fields, *Castoponera christae* sp. n. or *Castoponera lecythus* occur sympatrically with Ponerinae ants such as *Diacamma* Mayr, *Odontoponera* Mayr and *Leptogenys* Roger. These ants might be the suitable models of Batesian mimicry for *Castoponera christae* sp. n. and *Castoponera lecythus* because they are common and abundant in the forest floor, and have a sting. *Castoponera christae* sp. n. and *Castoponera lecythus* are especially similar to *Diacamma* spp. in the coloration and setation of the abdomen (Figs 36–37). The transversal bands of white setae on the abdomen emphasize the similarity to *Diacamma* ants.

## Supplementary Material

XML Treatment for Castoponera christae

XML Treatment for Castoponera lecythus
